# Discussions

**Published:** 1874-03

**Authors:** 


### DISCUSSIONS.

*Before the Eighth Annual Meeting of the Ohio State Dental Society, Dec. 4—6, 1873.*

What are the benefits to be derived from a proper care of the Deciduous Teeth, etc.

Dr. Watt: The benefits to be derived from a proper care of the deciduous teeth are many and various. The child as soon as it has teeth ought to begin to eat soft food. Childhood is usually precarious, but few children are healthy. During last summer, along about September, one-half of the deaths in Cincinnati were of children three years old and under. I suppose that the ordinary animals are of healthier organization. A colt is about as near being a horse when it is three months old as a child is to a man at three years. To take care of the teeth we will have to begin to raise healthy babies, of course care of the mother is important. The mother's care begins from the time the child begins intra-uterine growth. The child must come from a healthy womb, must have healthy blood, stomach, etc. The general constitution of children is wonderfully defective, or the majority would not die before they are three years of age, as they do. An eminent doctor of New York estimates that half the deaths of the human family occur before five years of age. We must have a radical revolution in this respect. We must begin to give attention. We must watch every point. We must have security against every defect. The child must masticate its food thoroughly and digest it properly. Satisfactory results will follow this. If food is not of the proper kind and properly digested, we have deleterious blood, and the health of the child destroyed for want of proper nourishment. I know one man who from the time his babies cut teeth rubs the gums morning and evening with prepared chalk on his finger. As soon as the children are old enough to use a soft brush he directs them to use it with prepared chalk. When the child is older it has become so habituated to the practice that it is second nature. The result in the case of these children so far is most satisfactory: they are most perfect models of children.

If the temporary teeth are permitted to decay, the mucous membrane becomes inflamed. You know how rapidly this inflammation extends. The mucous membrane of the stomach, etc., are all disturbed on account of diseased, or morbid condition of the mouth. This decay is sometimes the result of neglect. Disease is not an uncommon accompaniment of this morbid condition of the mouth, and I insist that we have no right to permit children to run any such risks.

Dr. Herriott: One of the great benefits to be derived from the proper care of the teeth is that children should be allowed the proper use of their teeth. If they are properly cared for, there is no trouble; if not they may be destroyed. The child should be taught to use its teeth.

Again, I differ with some of our professional brethren in regard to the importance of the first or temporary teeth. I must say that I am much surprised to find in a journal edited by a man who stands high in the profession, that he takes the ground that the first teeth were unnecessary in developing the jaw and its growth to enable the permanent teeth to come in their proper place. This I say was surprising to me and entirely contrary to that which I have always regarded as a necessity towards the preservation of the deciduous teeth.

Dr. J. Taft: There are many things involved in this question ; we can not discuss all the points involved. There are some things which are permanent, however, which should be considered. Some have been referred to.

The preservation of the permanent teeth is largely dependent upon the condition of the temporary teeth—upon the preservation of these in a state of health and usefulness. This is true for two or three reasons. In the first place, by the temporary teeth becoming diseased, the proper development and growth of the permanent teeth will be interrupted to a greater or less extent, and become more susceptible to disease. If the welfare of the permanent teeth, therefore, depends to any extent upon the temporary, it is certainly a very important matter that the temporary teeth should be preserved. If the mother who is charged with the education, cultivation and maintainence of her child, would accustom it to the use of care and cleanliness of the teeth the habit would be established and remain. The child from the time it is able to have an appreciation of the health and welfare of its organism ought to give attention to its teeth. Those who have such charges should be careful that they perform it. The child may be so impressed with the importance of this subject that it will be a matter of necessity that the teeth receive attention. The good accomplished does not cease with the temporary teeth, but extends through life. We are all creatures of habit; much that we do is simply by force of habit. If every mother appreciated the importance of these facts early and followed the indications, they would be more likely to be carried out through the life of the person than if commenced at a later period. Mothers do not care for these things sufficiently, but by and by when disease, pain and loss of organs take place, they will have more care. They will appreciate. If permitted to go on in neglect until ten, twelve or fourteen years of age the child, in all probability, will neglect the care of its teeth all the way through life. Much responsibility rests here with the dentist, he should educate the people. This is a part of his business, as certainly and clearly a part of his professional business to so educate, to so imbue the minds of all concerned that the best results may be secured. Such instructions would enable them to do their duty to those in their care. All of us are negligent of our duty in this respect. There is a duty here, resting upon every individual. Why is it that, when we find our fellow-men injured in other respects we put forth our effort to save them ? but here we simply pass on and give no warning voice. We are aware of the conditions which, if permitted to remain, must result in the destruction of the mouth. What is our duty ? Evidently, to put those in danger on their guard in time to avoid fatal results. Why do the majority of children die before they get their temporary teeth? A large proportion do die directly or indirectly from defective teeth. In all the animal kingdom none fail so utterly as man in rearing their young. Why is it ? Simply because we overlook such important matters. The mothers overlook it. The teeth are neglected. See the condition, and say who are responsible. Mothers in one sense, and those whose business it is to arouse their attention and give them knowledge, are responsible for it, and more responsible, perhaps, than the mothers. Get the mothers in the habit of taking care of temporary teeth and you will find a great improvement in the condition of the permanent teeth.

Dr. J. H. Warner: In regard to the proposition made by my friend, that neglect of the deciduous teeth may frequently result in death, I will say that twelve months ago a physician sent a patient to me for the purpose of having a tooth extracted. Upon examining the case, I found it was a second molar tooth that was involved. A great deal of swelling of the face and neck. The patient was nineteen years of age. The mouth was in such a situation that I could not open it. As soon as I could get the forceps in and by probing, I discerned that the exterior portion of the tooth had decayed very considerably. I sent him back to the doctor and told him to save the patient for me. Three or four days afterwards I saw the doctor and asked him how his patient was. "Did you not hear of his death ?" he asked. The patient had died of inflammation of the throat, produced, no doubt, by the condition of his teeth. I think it is the duty of all men practicing dentistry to call the attention, especially of mothers to the importance of preserving these teeth. How often have we seen them coming for the purpose of having the six year molars extracted. They will say that the patient has not shed that tooth, do not know it is the permanent tooth and do not appreciate the fact, that it is necessary to call the attention of the mother to the fact.

Dr. Clippinger: I have been interested in this discussion, and know I will derive benefit from it. If we wish to do good to our patients, we must first get them to appreciate the deciduous teeth. There is the great stumbling-block, I think, with a large portion of the people that come into the hands of the profession. I think that we cut our own throats when we suffer ourselves to remove any tooth, even though it be at great expense and trouble to the patient, who should keep it there as long as he can. Whenever you show a disinclination to save a tooth you cut short your argument in favor of saving the tooth. We can not be too careful in keeping ourselves consistent in this matter, instructing and impressing upon their minds the importance of caring for their teeth. It is of very little use for us to spend our skill, time and labor on the teeth, and then have the patient go away and say, that is all the tooth needs, and unless the patient himself will appreciate the fact that he is under obligations to take care of the teeth

When they do appreciate this they will secure the best skill and advice. We should be very slow to remove a tooth as long as we can save it. We should not extract it until we have exhausted every remedy within our reach.

Dr. Herriott: All recognize that the temporary teeth perform a certain function which is important to the child. Therefore it seems necessary that these teeth should be preserved as long as possible. How to do the thing that is the question. There is this one fact. We find a majority of parents bring in their dear little ones to us suffering with tooth ache. The first thing is, "My dear, the doctor won't hurt you at all." They come with a lie in their mouth, and attempt to deceive the child. There is nothing that is so painful to me, and I make it a point to undeceive the child at once. I teach them, however, in such a way as not to make trouble. I will do anything rather than remove the tooth. I have been watching the teeth of an interesting child for two years. When she came to me she had simply four incisors and two or three molars. The last time I saw her she was cutting six bicuspids. The child is eleven years old; but fortunately in this case the molars are very nearly in position. Now, I have another child who was badly salivated before she got her first molars. The first thing was to save all these six year molars, and the proper time coming for the appearance of the upper bicuspid, I found it appearing in an imperfect state; I pushed it off with my finger. The germ (?) of these teeth were disappearing by this salal, but I was mistaken so far as these bicuspids were concerned. It is our duty to save these temporary teeth; I in this case saved them so that they were useful to her until the time of their coming out.

Dr. C. R. Butler: No matter how far we may go back in the principles of pathology, etc., we find the human system diseased; if parents will bring their children to us imperfectly organized, we can do them little good. They may call upon us in vain to eradicate the conditions of imperfect organization. Each individual, in a matter of diseased teeth, must necessarily act for himself, by the exercise of his own judgment, that based upon observation and experience, whether it be right or wrong; and if we can by coming here correct our erroneous judgments and improve our mode of procedure, we thereby render to each other a greater service, and by that means are enabled to go back and render better service to those who need our aid. Now, it is known to some that (I shall speak simply of my own method of treating such cases, and if it shall serve any good purpose well; if not I simply say that it is the best I have at the present time, and if any one can suggest a better mode of practice none will be more gratified than myself,) I have adopted a mode of treatment of child patients. The child needs the deciduous teeth just as much for the purpose for which they were placed there as it subsequently needs the permanent teeth. We all know if these teeth are in condition not to be used for mastication, that the mouth as well as the general condition of the child is that of d sease. The food is imperfectly masticated, imperfectly insalivated and imperfect digestion is the result. You will see that I have simply indicated the effect of this neglect on the general system. And this is where, perhaps, we make many mistakes, in simply looking at it in a partial manner, without reflecting that local disease may have produced certain effects on the general system, and by them certain results must be brought about in the order of proper functional action. There is much to be done in the care of them, in instructing the patient what is necessary to be done for the care of the teeth, in getting them in the habit of taking care of them. Now, much may be done to keep them along in comfortable shape while the temporary teeth are in the mouth; but when the first molars and the permanent teeth begin to make their appearance a great deal of damage is done by the dilapidated condition of the temporary teeth; so that the care of the temporary in a perfect manner, or as well as they may be cared for, becomes of great importance. Now, a few words in reference to my mode of managing this class of cases. I say to the patient, I will do the best I can if you will give me an opportunity. I want to see this child once, twice or thrice a month. Now, I want you to understand that you are to do for it the best you can, and let it come or bring it. They fail to do that.

Perhaps you won't see the child again until it has an exposed pulp or abscess or something of that kind. The child is bad. You say, if you had followed my instructions some of the trouble at least might have have been avoided. I say, now, if you want to put this child in my care you must give me an opportunity to take care of it, or else I will not be responsible for the results. I send by some of the family a line, perhaps, through the post-office; I want you to send this one or that to me and let me see it. Information comes that they will do it and thank you for it, too. If they are negligent about it I will not be responsible for this case at all if you propose to go on in this way. You have a right to say that. This giving lectures may be done a great many times, and a few words said kindly and earnestly will be remembered, and thereby you may do a valuable service. In this way we may also relieve ourselves of a great deal of inconvenience, besides doing the child a great service. Such discussion will often be productive of much good; try it. Talk properly and intelligently to children. They have sense and will appreciate good suggestions. They are in the plastic state and may be manipulated. Then here is another thing, I will give this as a sort of illustration of how we may reach them by indirect means. Here is a woman, she says: "Doctor, how are all my front teeth getting lose; what is to be done?" Oh, well nothing, you have lost all your back teeth. There is a condition, I say, perhaps that you never have met, but there is a direct demonstration of what follows the loss of the back teeth.. There are a thousand ways we may reach these cases, and these remarks that I have made I have endeavored to make as practicable as possible. I have heard it recommended here that the pulp of deciduous teeth may be destroyed. Now, I would most earnestly say, do not apply arsenic to the pulp of any deciduous tooth. Do not do it again if you have ever done it. Let nature take care of it rather than put in any of these poisons.

Dr. Herriott: I think that a great deal of the fault that is attributed to the children is the fault of the dentist. I commenced, in Zanesville, from the first to educate the people to give attention to the deciduous teeth, by the publication oarticles in the newspapers for which I was thanked by mothers and fathers. Those who were children then have not only taken care of their first teeth, but by habit they give attention to those that decay, and I venture that there are fewer nerves destroyed there by filling than elsewhere. A great deal of this fault is due to the dentist. It is a duty of every dentist to publish short-pointed articles in the newspapers to go into the hands of mothers for their instruction.

